# Effects of Pulsed 2.856 GHz Microwave Exposure on BM-MSCs Isolated from C57BL/6 Mice

**DOI:** 10.1371/journal.pone.0117550

**Published:** 2015-02-06

**Authors:** Changzhen Wang, Xiaoyan Wang, Hongmei Zhou, Guofu Dong, Xue Guan, Lifeng Wang, Xinping Xu, Shuiming Wang, Peng Chen, Ruiyun Peng, Xiangjun Hu

**Affiliations:** 1 Beijing Institute of Radiation Medicine, Beijing, China; 2 Beijing Institute of Basic Medical Sciences, Beijing, China; 3 NO. 281 Hospital of People’s Liberation Army, Qinhuangdao, China; Instituto Butantan, BRAZIL

## Abstract

The increasing use of microwave devices over recent years has meant the bioeffects of microwave exposure have been widely investigated and reported. However the exact biological fate of bone marrow MSCs (BM-MSCs) after microwave radiation remains unknown. In this study, the potential cytotoxicity on MSC proliferation, apoptosis, cell cycle, and in vitro differentiation were assayed following 2.856 GHz microwave exposure at a specific absorption rate (SAR) of 4 W/kg. Importantly, our findings indicated no significant changes in cell viability, cell division and apoptosis after microwave treatment. Furthermore, we detected no significant effects on the differentiation ability of these cells in vitro, with the exception of reduction in mRNA expression levels of osteopontin (OPN) and osteocalcin (OCN). These findings suggest that microwave treatment at a SAR of 4 W/kg has undefined adverse effects on BM-MSCs. However, the reduced-expression of proteins related to osteogenic differentiation suggests that microwave can the influence at the mRNA expression genetic level.

## Introduction

Mesenchymal stem cells (MSCs) are multipotent cells that can be induced to differentiate into a variety of mesenchymal tissues, including bone, cartilage, fat, bone marrow stroma, and muscle [1–3]. Despite being widely used in cell-based therapy and tissue engineering, the fate of MSCs after microwave exposure is largely unknown. Microwaves exist at a rate of oscillation of an electromagnetic field in the range of approximately 300 MHz to 300 GHz. They are considered to be a non-ionizing electromagnetic field and are typically present in the environment through radar, radio/TV communications, mobile-phone base stations, occupational use, and medical applications [4–6]. Therefore, the influence of 2.856 GHz microwaves at the cellular and molecular level is a critical area of research, which may provide insights into the appearance of common genotoxic effects that are yet to be resolved.

Biophysical stimulations such as pulsed electromagnetic field have been extensively employed in clinical settings to accelerate and finalize the healing process of a fresh fracture, and to enhance the spontaneous repair capability of bone tissue [7–9]. Several reports have indicated that pulsed electromagnetic field may play a key role in affecting the differentiation ability of mesenchymal stem cells (MSCs) [10–12]. However, to the best of our knowledge, a systemic study to evaluate the potential effects of pulsed 2.856 GHz microwave on MSCs in vitro has not been performed. Generally, radiofrequency/microwave radiation is classified as an environmental pollutant that can be harmful to human health [13]. Although it may not directly cause DNA damage (strand deterioration), several adverse effects on multiple targets after microwave exposure have been reported [5,14,15]. Our previous findings have revealed that non-ionizing radiation electromagnetic pulses (EMP) are indeed unable to induce oxidative stress but reduce the generation of free radicals in rat liver mitochondria [16]. In 2011, the International Agency for Research on Cancer (IARC) classified radiofrequency electromagnetic fields as “possibly carcinogenic to humans” (Group 2B) [13]. This has often been misinterpreted as indicating that some measure of risk has been observed. To date, research has suggested that the possible adverse effects on human cannot be conclusively ruled out based on the available data [13,17,18]. However, strong evidence has been shown in recent studies that the long-term usage of mobile and cordless phones is correlated with cancer risk [19–23]. In this study, MSCs isolated from C57BL/6 mice bone and bone marrow were treated with pulsed 2.856 GHz microwave with a SAR level of 4 W/kg. The SAR was chosen according to the American National Standard Institute (ANSI) standards for safe exposure levels to microwave radiation [24]. Consequently, the potential cytotoxicity on MSCs proliferation, apoptosis, cell cycle, and in vitro differentiation were assayed.

## Materials and Methods

### Mice

Male C57BL/6 mice (age 2.5 weeks; permission number: SCXK-2007-004) were provided by the Laboratory Animal Center, Academy of Military Medical Sciences, Beijing, China. Six mice were housed in accordance with institutional animal care policies with access to water and food under the standard laboratory procedures. All experiments were performed under protocols approved by the Academy of Military Medical Sciences Animal Care and Use Committee and from the approval of our ethics committee.

### Cells

Primary murine MSCs derived from murine bone and bone marrow were isolated and cultured as described previously [25]. Briefly, the mice were sacrificed by cervical dislocation and their bilateral femurs and tibias were retrieved and ground under sterile conditions. After digestion with collagenase II and centrifugation, the bone pieces and pellets were seeded and grown in minimal essential medium (MEM, Gibco) consisting of 4 mM L-glutamine, 100 U/mL penicillin, 100 U/mL streptomycin, and 10% fetal bovine serum (FBS). The medium was changed 72 h after seeding and then subsequently every 3 days. When the cells coated approximately 70% of the bottom of the bottle, they were digested with 0.25% trypsin, diluted and then subcultured. The cells were cultured in a humidified atmosphere of 5% CO_2_ at 37°C.

### Experimental groups

For detection of MSCs proliferation, apoptosis and cell cycle, the MSCs from a single mouse were divided into three groups: (i) sham, (ii) microwave exposure and (iii) positive control which was irradiated with 2.0 Gy ^60^Co γ-ray. Each group contained at least three samples or Petri dishes from three different mice. The MSCs isolated from one mouse were divided into two groups: (i) sham and (ii) microwave exposure for detection of in vitro differentiation and mRNA expression for OCN and OPN, each group contained at least three samples from another three mice.

### Pulsed microwave exposure and γ-ray radiation

**Fig 1:**
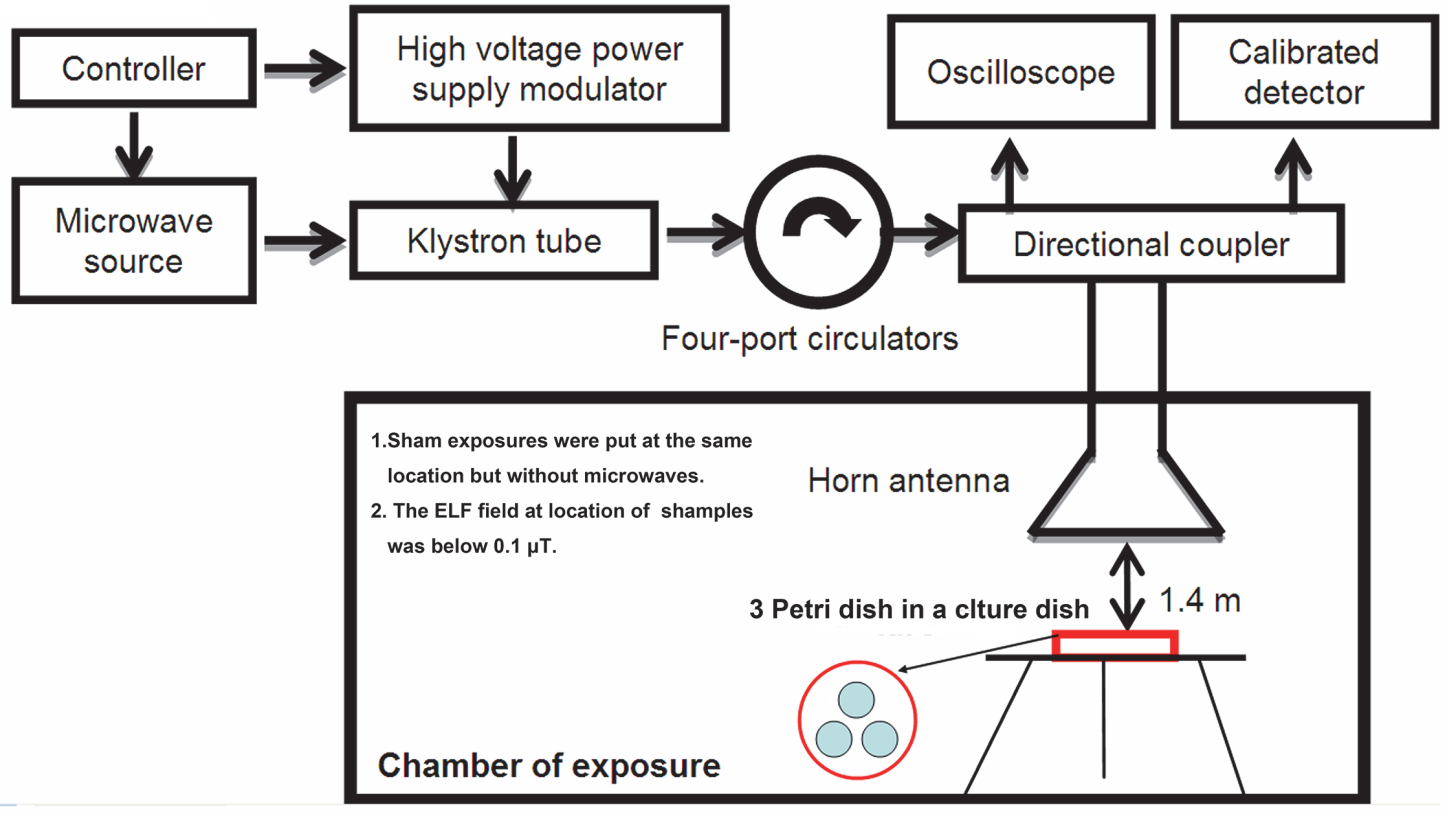
Schematic diagram of the microwave exposure system which mainly comprises the microwave source, klystron tube, four-port circulators, directional coupler, and horn antenna.

A previously described microwave exposure system was used in this study [26]. In brief, 2.856 GHz pulsed microwaves were generated and transmitted by a microwave source and rectangular waveguide, and microwave energy was transmitted to an electromagnetic shield chamber by an A16-dB standard-gain horn antenna for MSCs exposure. The distance from the antenna to the top of the culture dish was 1.4 m (Fig. 1). The microwave pulses were delivered at 50 pulses per second (pps), with a pulse width of 500 ns. The peak field power densities were tested with a calibrated detector and the oscilloscope was set to 200 W/cm^2^. The average field power densities were calculated as 5 mW/cm^2^, and the exposure time was set to 6 min. The relative dielectric constant of the culture medium was 76.4, and the conductivity was 2.5 S/m. The density of the medium was equal to that of water. The SAR was calculated to be 4 W/kg, based on the finite difference time domain (FDTD) method using the following formula: SAR = σE^2^/ ρ(W/kg) [27,28]. The sham exposure group was handled and processed in parallel to the exposure groups but without exposure to microwaves. The ELF background fields at the location of sham and microwave exposures were below 0.1 μT detected with ELF fields strength measurement system (Holaday Industrial Ins. HI-3604), the static magnetic fields were mainly came from the natural static magnetic fields of the earth. At the same time, the positive controls were irradiated with ^60^Co γ-ray in the radiation center of Beijing Institute of Radiation Medicine with a dose rate of 1.0 Gy/min for 2.0 min.

### Monitoring temperature

**Fig 2:**
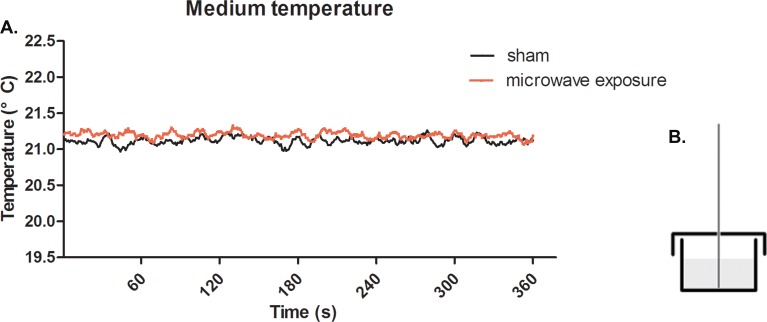
Real-time temperature detection of medium under 2.856 GHz microwave exposure at SAR of 4 W/kg. (A) Solid red line represents the temperature changes under microwave exposure and the solid black line represents the sham group in parallel to the exposure group (in the absence of microwave radiation). (B) Schematic diagram describing the geometry of temperature measurements.

The real-time variation of the culture medium temperature was monitored using an optical fiber temperature probe (interval time: 1.0 s, resolution: 0.1°C) and a M3300 optical fiber thermometer (Luxtron Co., Santa Clara, CA., USA). The geometry of the temperature measurement is displayed in Fig. 2B. The diameter of the temperature probe was 0.5 mm, and the distance from the probe to bottom of culture dish was 0.5 mm.

### MTT assay

Briefly, 100 μL BM-MSC cells (4×10^3^ per well) were seeded in 96-well plates immediately after microwave exposure. The microwave and sham exposure groups were cultured on the same plate, with four plates in total. Cell proliferation was detected at days 1, 2, 4, and 6 after exposure. Then, 20 μL MTT (5 mg/mL dissolved in PBS) was added to each well and incubated at 37°C for 4 h. Subsequently, the supernatant was completely removed and 150 μL DMSO was added to the solution, followed by vigorous shaking for 10 min. Absorbance was detected at 490 nm with ELLASA (Multiskan MK3, Thermo Co. USA), where the OD_490nm_ represented the activity of cell viability.

### Flow cytometry

MSCs were collected at 6 h after microwave exposure and washed twice with PBS. Apoptosis and cell cycle were assayed according to the manufacturer’s instructions using commercially available colorimetric kits (Nanjing KeyGEN Biotech. Co. Ltd., China). Data were collected on a FACS Cytomics FC-500 (Beckman Coulter) and analyzed using CXP 2.1 software (Beckman Coulter).

### In vitro differentiation

For in vitro differentiation, cells were induced with osteogenic induction media containing 0.1 μM dexamethasone, 50 μM ascorbate-2 phosphate，and 10 mM glycerophosphate (Sigma). To induce adipogenic differentiation, cells were cultured in adipogenic induction media containing 1 μM dexamethasone, 200 μM indomethacin, 0.5 μM 3-isobutyl-1-methyl-xanthine, and 10 μg/mL insulin (Sigma). Von Kossa and Oil-Red-O staining were performed as described previously to characterize osteoblastss and adipocytes [25,29]. To induce chondrogenic differentiation, a commercial chondrogenesis differentiation kit was employed according to the manufacturer’s protocols (Gibco).

### Differentiated chondrogenic pellet histopathology

H&E staining was performed to analyze chondrocyte differentiation. The differentiated chondrogenic pellets were fixed in 10% (v/v) neutral formalin, embedded in paraffin and sectioned into 5-μm slices. After dewaxing, the sections were stained with hematoxylin for 5 min and eosin for 60 s. Further microscopic examinations were performed after the slices were mounted.

### Real-time PCR

Total RNA was extracted with Trizol (Sigma) and reverse-transcribed into cDNA using a reverse transcriptase kit (Takara). cDNA was used as the template for real-time PCR (StepOne Real-Time PCR system, Applied Biosystems. Inc., USA) with SYBR-Green reagent (Applied Biosystems. Inc.) to determine specific gene expression. Primer sequences were as follows: mouse β-actin, 5′-GGCCCAGAGCAAGAGAGGTA-3′ (forward) and 5′-CATGTCGTCCCAGTTGGTAACA-3′ (reverse); mouse OPN, 5′-AGCAAGAAACTCTTCCAAGCAA-3′ (forward) and 5′-GTGAGATTCGTCAGATTCATCCG-3′ (reverse); mouse OCN 5′-CTGACCTCACAGATCCCAAGC-3′ (forward) and 5′-TGGTCTGATAGCTCGTCACAAG -3′ (reverse). The PCR reaction conditions were 95°C for 10 min, 40 cycles of 95°C for 15 s, 60°C for 15 s, and 72°C for 40 s during the holding and cycling stages, and finally 95°C for 15 s, 60°C for 1 min, and 95°C for 15s during the melt curve stage.

### Statistical analysis

The experiments were blind-designed with experiment grouping, detections and statistical analysis performed by different authors. Data are presented as the mean ± SD. Statistical differences were assessed using the unpaired two-tailed Student’s *t*-test and one-way analysis of variance (ANOVA). Differences at *P* < 0.05 were considered significant. Statistical power was estimated and calculated using software SAS 9.1.3.

## Results

### Microwave exposure effect on medium temperature

Microwave energy was emitted by an A16-dB standard-gain horn antenna to an electromagnetic shield chamber (dimensions, 7×6.5×4 m; detailed description provided in a previous report [26]). The temperature and humidity of the chamber was controlled throughout the measurements. The cell samples were placed under the antenna with a working distance of 1.4 m. Upon examination, we found no apparent changes in temperature when the samples were exposed to microwaves. In the case of the sham exposure groups, the temperature was found to remain at approximately 21°C within a 6 min period (Fig. 2A). The results indicated that the microwave at SAR of 4 W/kg had no effect on the medium temperature, indicating that there were non-thermal effects when the SAR settings below 4 W/kg.

### MSC proliferation viability unaffected by microwave exposure

**Fig 3:**
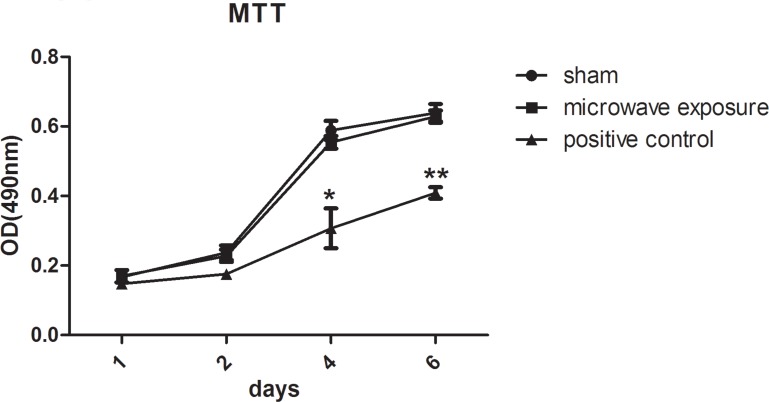
MSC proliferation detected with MTT assays after 2.856 GHz microwave exposure at SAR of 4 W/kg (n = 3, each group contained three cell samples from three different mice). Solid line with black squares represent the microwave exposure group, solid line with points represent the sham group in parallel to the exposure group in the absence of microwave radiation, and the solid line with black triangles represent the positive control irradiated with 2.0 Gy ^60^Co γ-ray. Compared with sham group, cell viability decreased significantly at day 4 and day 6 with the OD_490nm_ values of 0.307 ± 0.058 (n = 3, *P* = 0.023, statistical power ＞0.999) and 0.409 ± 0.016 (n = 3, *P* = 0.001, statistical power ＞0.999) after γ-ray radiation. However, there were no significant differences in cell viability between sham and microwave exposures.

MTT assays were conducted at days 1, 2, 4, and 6 after microwave exposure. MSC proliferation was found to increase significantly within 6 days in sham and exposure groups, the OD_490nm_ values in the sham and exposure groups increased from 0.167 ± 0.01 to 0.639 ± 0.025 (n = 3) and from 0.169 ± 0.017 to 0.628 ± 0.017 (n = 3), respectively, resulting in good levels of cell viability (Fig. 3). There were no obvious differences between sham and exposure groups with *P* value of 0.84 (statistical power = 0.052) and 0.33 (statistical power = 0.07). Moreover, MSC physiological conditions presented no abnormality after exposure compared to the sham group, as observed using optical microscopy. However, cell viability decreased significantly compared to sham group at day 4 and day 6 with the OD_490nm_ values of 0.307 ± 0.058 (n = 3, *P* = 0.023, statistical power ＞0.999) and 0.409 ± 0.016 (n = 3, *P* = 0.001, statistical power ＞0.999) after γ-ray radiation (Fig. 3). The MTT assay results indicated that 2.856 GHz pulsed microwave treatment at SAR of 4 W/kg did not influence the MSC viability and proliferation characteristics.

### Effect of pulsed microwave on MSC apoptosis

**Fig 4:**
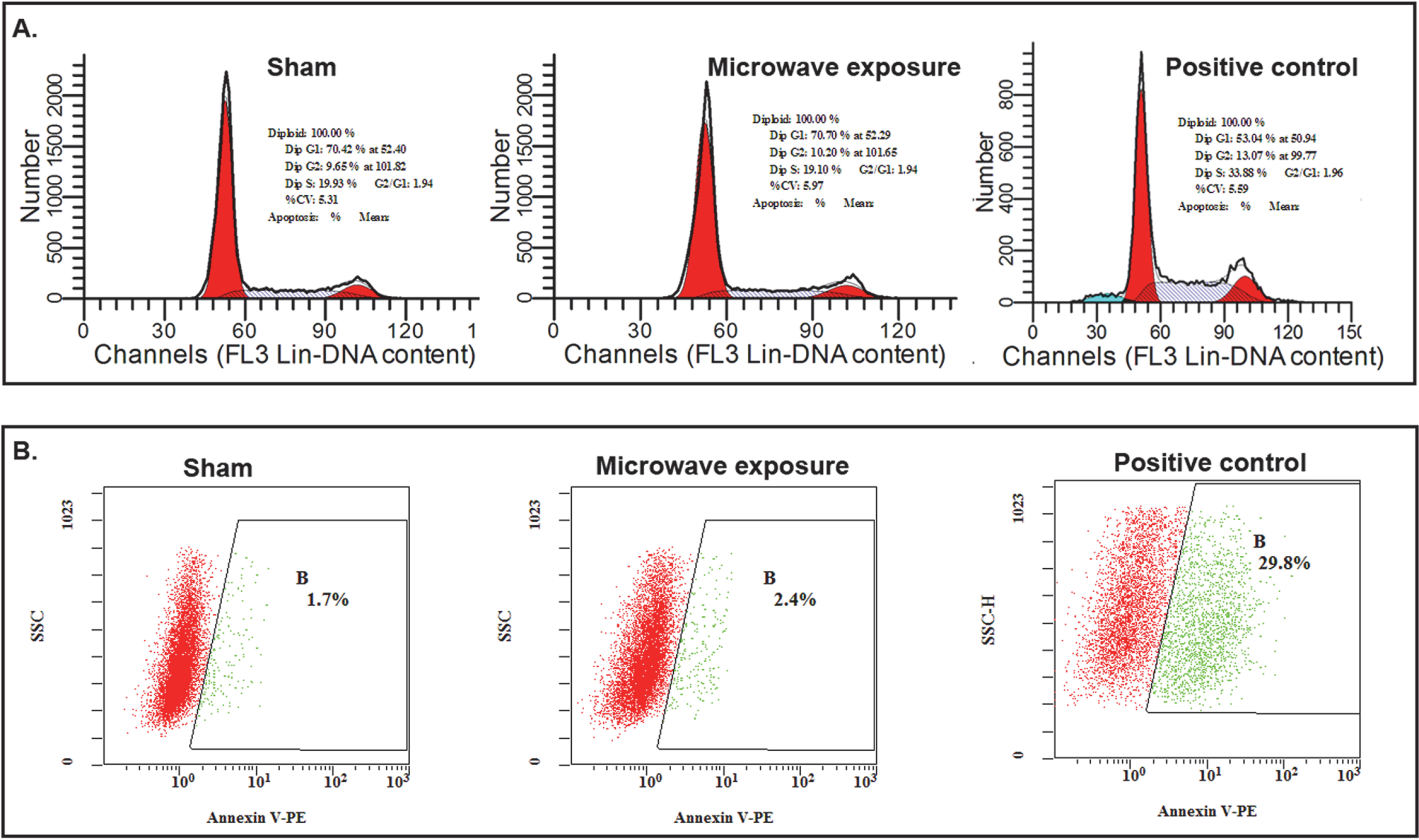
Cell cycle and apoptosis of MSCs detected with flow cytometry after 2.856 GHz microwave exposure at SAR of 4 W/kg. (A) Cell cycle detected with PI. The representative results of sham, microwave exposure and the positive control were shown in the upper panel. (B) Apoptosis detected with annexin V-PE. The representative results of sham, microwave exposure and the positive control were shown in the lower panel.

Apoptosis and cell cycle were detected at 6 h after exposure. Representative results are shown in Fig. 4. The cell cycle results showed that cells from the both sham and exposure groups were predominantly located in the G1 stage, exhibiting good levels of viability (Fig. 4A): sham exposure, 70.42% cells at 52.4 and exposure, 70.7% at 52.29 (Fig. 4A). In the G2 phase, 9.65% of cells in the sham exposure group were detected at 101.8 and 10.20% of those in the exposure group at 101.65 (Fig. 4A). Overall, 19.93% cells of the sham group were in the S phase compared to 19.10% of the exposure group (Fig. 4A). The results indicated that the 2.856 GHz pulsed microwave treatment at SAR of 4 W/kg was not able to induce MSCs apoptosis. However, in the γ-irradiated group 53.04% cells were at 50.94 and 13.07% of those at 99.77 in the G1 and G2 phase respectively, 33.88% cells were at S stage indicating the decreased cell viability. The results were further confirmed using an annexin V-PE apoptosis detection kit (Nanjing KeyGEN Biotech. Co. Ltd., China). In this case, the apoptosis rate of the sham and exposure groups were estimated at 2.67% ± 0.55% (n = 3) and 3.07% ± 0.49% (n = 3), respectively, displaying minimal variation after microwave exposure without obvious statistical differences with *P* value of 0.55 (statistical power = 0.113). However, ^60^Co γ-ray radiation obviously induced apoptosis with the value of 28.4% ± 3.89% compared with sham group (n = 3, *P* = 0.009, statistical power ＞0.999). Representative results are shown in Fig. 4B.

### In vitro differentiation of MSCs and osteogenic protein mRNA expression

**Fig 5:**
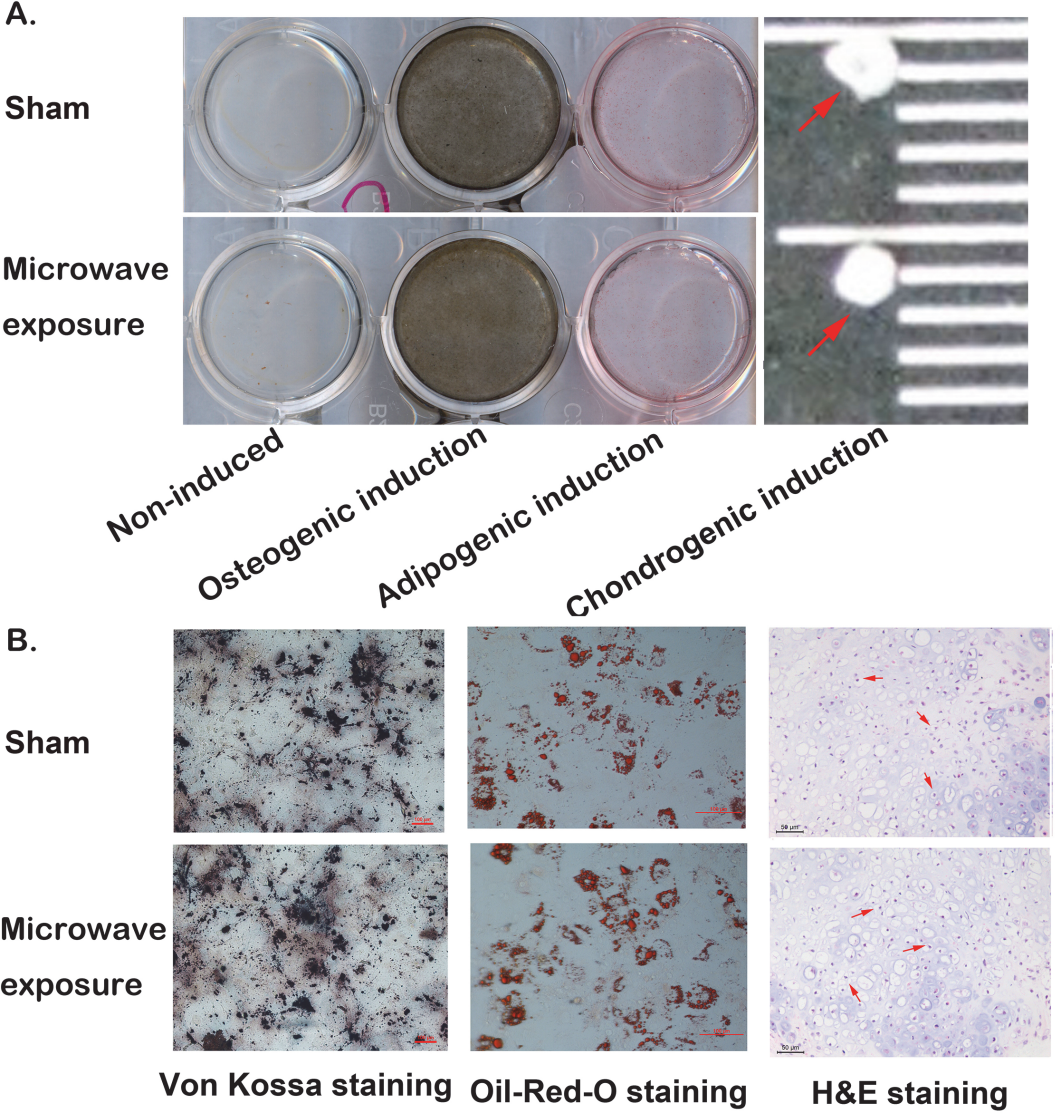
MSCs in vitro differentiation after 2.856 GHz microwave exposure at SAR of 4 W/kg. (A) Osteogenic induction and adipogenic induction were detected with Von Kossa and Oil-Red-O staining. The induced chondrogenic pellets were placed onto a black ruler as indicated by the arrows. (B) Microscopic examination of induced osteoplasts (*left panel*), and adipocyted (*middle panel*) with Von Kossa and Oil-Red-O staining (scale bar = 100 μm), and chondrocytes (*right panel*) with H&E staining (scale bar = 50 μm).

**Fig 6:**
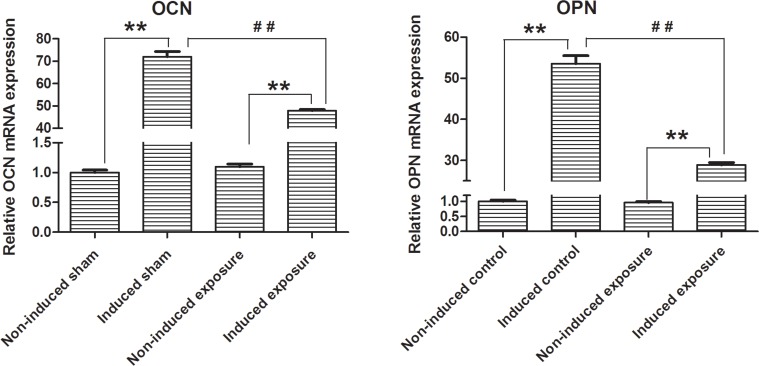
Relative OPN and OCN mRNA expression detected with Q-PCR after 2.856 GHz microwave exposure at SAR of 4 W/kg. (*left panel*) OCN; (*right panel*) OPN. Compared with the non-induced groups, mRNA levels of OCN and OPN were both significantly increased after induction (**, n = 3, *P* < 0.01, statistical power ＞0.999, each group contained three samples from three different mice). OPN and OCN mRNA expression levels were both significantly reduced after microwave exposure compared with the sham group (^##^, n = 3, *P* < 0.01, statistical power ＞0.999, each group contained three samples from three different mice).

MSCs were induced with different induction media immediately after microwave exposure. Osteoblasts, adipogenic, and chondrogenic cells were differentiated from MSCs in both the sham and microwave exposure groups. However, we found no significant differences between the two groups (Fig. 5). Von Kossa and Oil-Red-O staining demonstrated that high amounts of calcium and fat were deposited and secreted after induction across the two groups, and there were no obvious differences in average optical density between two groups assayed with software LeicaV3.4 (Fig. 5B). The average optical densities of Von Kossa staining in sham and exposure groups were 52.83 ± 4.65 (n = 3) and 51.54 ± 2.50 (n = 3), respectively. For Oil-Red-O staining, average optical densities in both groups were 20.51 ± 1.65 (n = 3) and 21.17 ± 2.60 (n = 3), respectively. There were no statistical differences in osteogenic and adipogenic differentiations between two groups with *P* values of 0.74 (statistical power = 0.063) and 0.81 (statistical power = 0.060). Concurrently, there were no distinct changes in the size of induced chondrogenic pellets (Fig. 5A). Furthermore, H&E staining showed that chondrogenic cells in both cases were arranged evenly as shown by the arrows in Fig. 5B. The relative osteogenic proteins, OCN and OPN were detected using qPCR. Compared with non-induced groups, OCN and OPN mRNA levels were both significantly increased after induction (n = 3, *P* < 0.01). Interestingly, these results indicated that the induced mRNA expression levels of OPN and OCN were both significantly reduced after microwave exposure compared with the induced sham groups. The relative induced mRNA expression level of OCN and OPN decreased significantly from 71.94 ± 2.31 and 53.52 ± 1.97 to 47.89 ± 0.49 and 28.90 ± 0.57 after exposure, respectively. (n = 3, *P* < 0.01, statistical power ＞0.999) (Fig. 6).

## Discussion

Our results indicate that BM-MSCs exposed to 2.856 GHz microwave radiation at a SAR of 4 W/kg displayed no significant changes in cell viability, apoptosis, and in vitro differentiation, with the exception of reduced levels of mRNA expression for OCN and OPN.

With the ever-increasing application of microwaves, public concerns about their possible health impact have been raised. Several studies have shown that microwaves cause various biological effects depending upon their field strengths, frequencies, waveforms, modulation and durations of exposure [30,31]. Such microwave-induced damage is known to lead to death in single cell organisms, inhibit cell proliferation, cause DNA damage and alter gene expression [32–35]. However, no obvious adverse effects after the exposure of MSCs to microwaves were observed in this study.

We observed no changes in medium temperature, which suggested a non-thermal effect on MSCs after microwave exposure. Our results indicated that microwave exposure had no effect on MSC proliferation, apoptosis, and differentiation. Nevertheless, the expression levels of OCN and OPN mRNA were reduced after exposure, which suggests that microwaves may influence MSCs at the level of transcription.

Microwaves from mobile phones may inhibit the formation of 53BP1 foci in human primary fibroblasts, MSCs, and lymphocytes, which indicates a possible links to cancer risk [36]. However，the pre-exposure of mice to 900-MHz radiofrequency fields showed that adaptive responses reduce the level of hematopoietic damage caused by ionizing radiation [37–39]. The differentiation of MSCs into chondrocytes could be enhanced by millimeter waves [40]; however the fact that 2.856 GHz microwaves had no effects on in vitro differentiation capabilities may have been the result of different wave types and irradiation conditions. In addition, microwaves may alter cell morphology, disrupt cell division [41], and influence cell membrane permeability [42], resulting in either the degeneration, apoptosis or necrosis of cells at different stages. Recent work on chromosomes and DNA as targets for resonance interaction between living cells and microwaves [43] had also suggested that exposure to microwave radiation can damage the gene structure [44]. Meanwhile, numerous experimental evidences have reported that radiofrequency radiation does not induce genetic effects [45,46].

Thus, identifying and evaluating the biological effects of microwave has been a complex and controversial process because the mechanisms by which microwave exert their effects remain poorly characterized. It is critical that the key targets and mechanisms of pulsed microwaves should be characterized in the near future.
